# Management of concomitant cholecystoduodenal and cholecystocolonic fistulas with sigmoid colonic gallstone: a case report

**DOI:** 10.1093/jscr/rjag866

**Published:** 2026-09-29

**Authors:** Akinfemi Akingboye, Sadik Al-Hassani, Devyani Bhatt, Adrian Hall, Chaminda Sellahewa

**Affiliations:** Department of General Surgery, The Dudley Group Foundation NHS Trust, Pensnett Road, Dudley, West Midlands, DY1 2HQ, United Kingdom; College of Health and Life Science, Aston Medical School, Aston University, Aston Triangle, Birmingham, B4 7ET, United Kingdom; Department of General Surgery, The Dudley Group Foundation NHS Trust, Pensnett Road, Dudley, West Midlands, DY1 2HQ, United Kingdom; Department of General Surgery, The Dudley Group Foundation NHS Trust, Pensnett Road, Dudley, West Midlands, DY1 2HQ, United Kingdom; Department of General Surgery, The Dudley Group Foundation NHS Trust, Pensnett Road, Dudley, West Midlands, DY1 2HQ, United Kingdom; Department of General Surgery, The Dudley Group Foundation NHS Trust, Pensnett Road, Dudley, West Midlands, DY1 2HQ, United Kingdom

**Keywords:** cholecystoenteric, fistula, cholecystoduodenal, cholecystocolonic, gallstone

## Abstract

Cholecystoduodenal fistula and cholecystocolonic fistula are rare complications of gallstone pathology. A 62-year-old female presented to our surgical assessment unit with abdominal pain and vomiting. A computed tomographic scan revealed complex biliary-enteric fistulas. The fistulas involved the gallbladder, duodenum, and colon, with evidence of partial gastric outlet obstruction secondary to a gallstone in the proximal duodenum. Furthermore, imaging revealed a large gallstone impacted proximal to the diverticular stricture in the sigmoid colon. A minimally invasive approach to resolve the fistula was unsuccessful; endoscopic retrograde cholangiopancreatography and sigmoidoscopy to dislodge the gallstones failed. This led to a laparotomy with jejunal enterotomy for stone removal and a Hartmann’s procedure to address the impacted gallstone and the sigmoid diverticular stricture. Furthermore, the repair of both fistulae was performed during the index operation at the second operation, while dealing with unexpected colostomy necrosis.

## Introduction

Cholecystoenteric fistulas are rare complications of chronic biliary disease. Cholecystoduodenal fistula (CDF) is the most common type of cholecystoenteric fistula, followed by cholecystocolonic fistula (CCF). They commonly present with nonspecific symptoms such as diarrhoea and abdominal pain. This type of fistula can be further complicated by causing a gastric outlet obstruction secondary to gallstones, known as Bouveret syndrome [1]. Furthermore, abdominal X-ray and computed tomography (CT) may show a combination of signs known as Rigler’s triad (pneumobilia, ectopic gallstones, and intestinal obstruction) [1]. Due to the nonspecific symptoms and rarity of the condition, preoperative diagnosis may be difficult; hence, the invaluable role of CT of the abdomen and pelvis in the management of acute surgical emergencies.

## Case presentation

A 62-year-old obese Caucasian woman presented to the emergency department with a 3-week history of diarrhoea, vomiting, increased bowel frequency (9–10 times daily), and colicky epigastric pain migrating to the left iliac fossa, without rectal bleeding. Her medical history included hypertension, cholelithiasis, gastro-oesophageal reflux disease, and obesity (BMI 38 kg/m^2^). She had previously undergone a total abdominal hysterectomy for uterine fibroids and was classified as ASA grade II. Initial full blood count and liver function tests were normal. She was discharged with an urgent outpatient colonoscopy appointment but was readmitted two days later because of worsening symptoms.

On readmission, C-reactive protein was markedly elevated at 264 mg/L despite a normal white cell count. CT demonstrated a 3.1-cm gallstone fistulating into the first part of the duodenum (Figs 1 and 2), a 3.5-cm impacted gallstone within the distal sigmoid colon (Figs 2 and 3), and a complex fistulous communication involving the gallbladder, duodenum, and colon (Fig. 4). Concomitant sigmoid diverticulitis and a suspected stricture distal to the impacted stone were identified. Initial differentials included gastric outlet obstruction and colitis. CT demonstrated no suspicious bowel lesions or mural thickening. Intravenous antibiotics were commenced for presumed intra-abdominal sepsis secondary to acute diverticulitis or cholecystitis.

**Figure 1:**
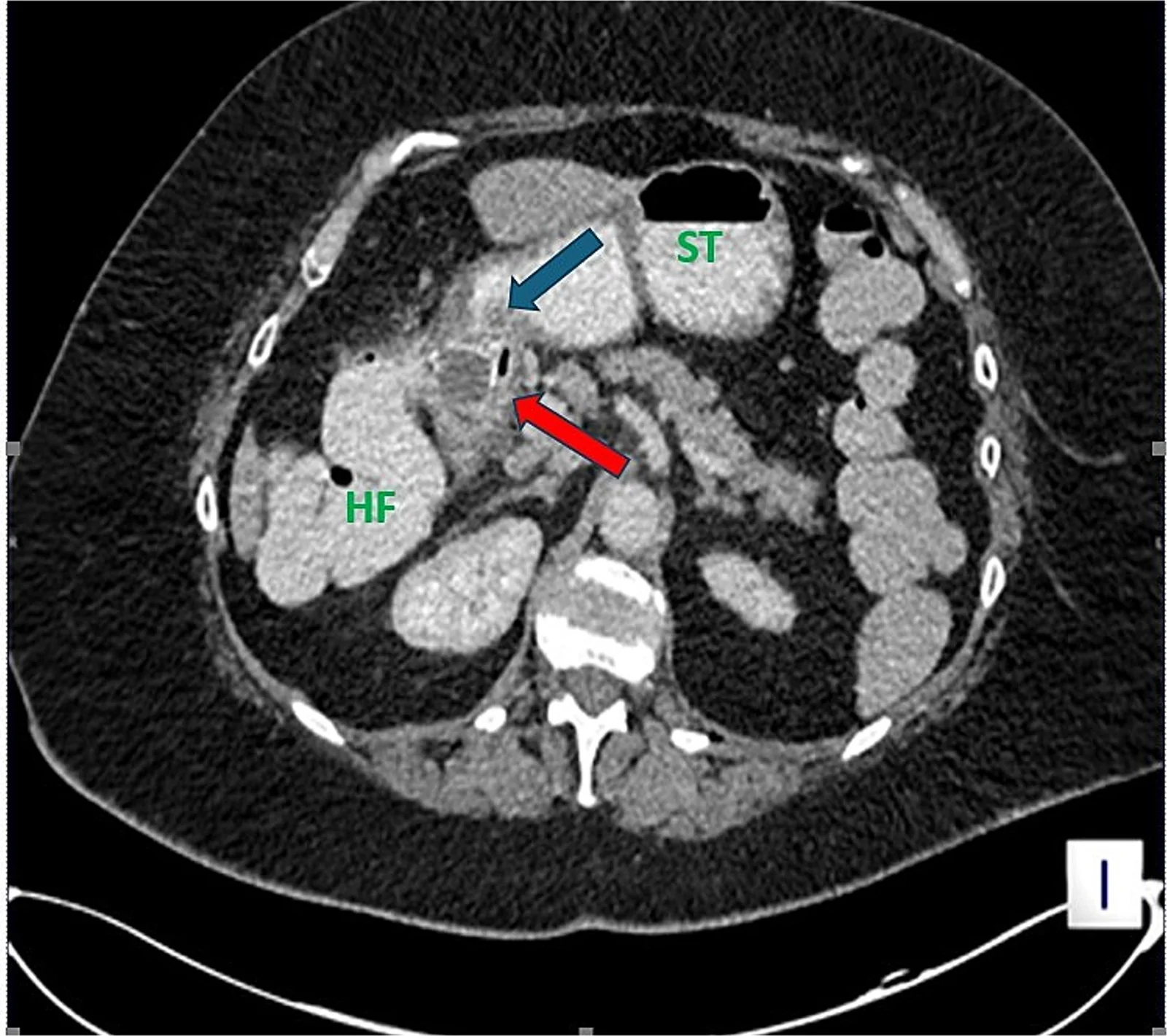
Axial CT scan with IV and oral contrast. Hyperdense oral contrast opacifies the stomach (ST) and hepatic flexure (HF). A round gallstone is seen in the proximal duodenum (red arrow).

**Figure 2:**
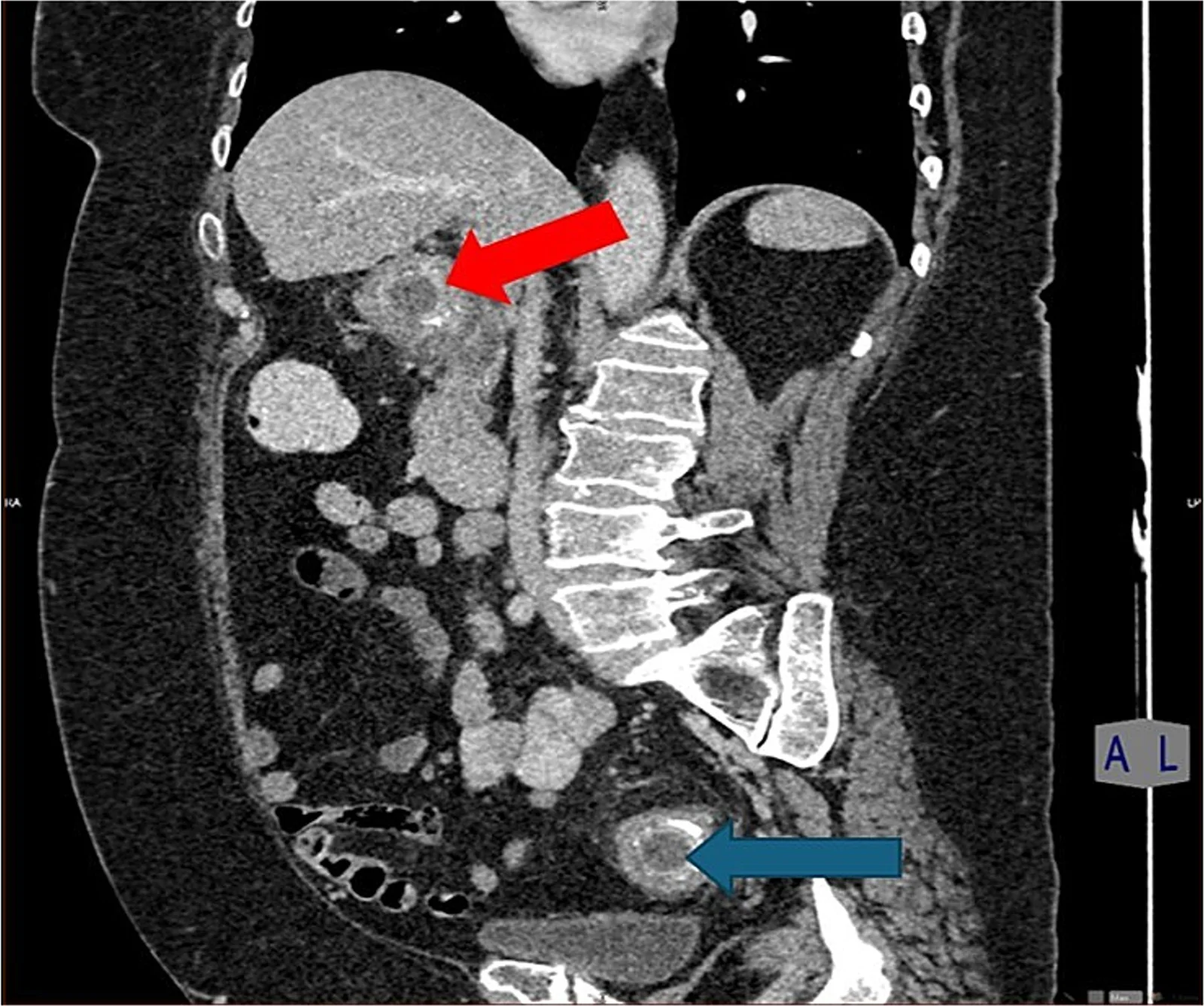
Sagittal reconstruction CT image. Round low-density gallstones showing some rim calcification are seen in the proximal duodenum (red arrow) and the sigmoid colon (blue arrow).

**Figure 3:**
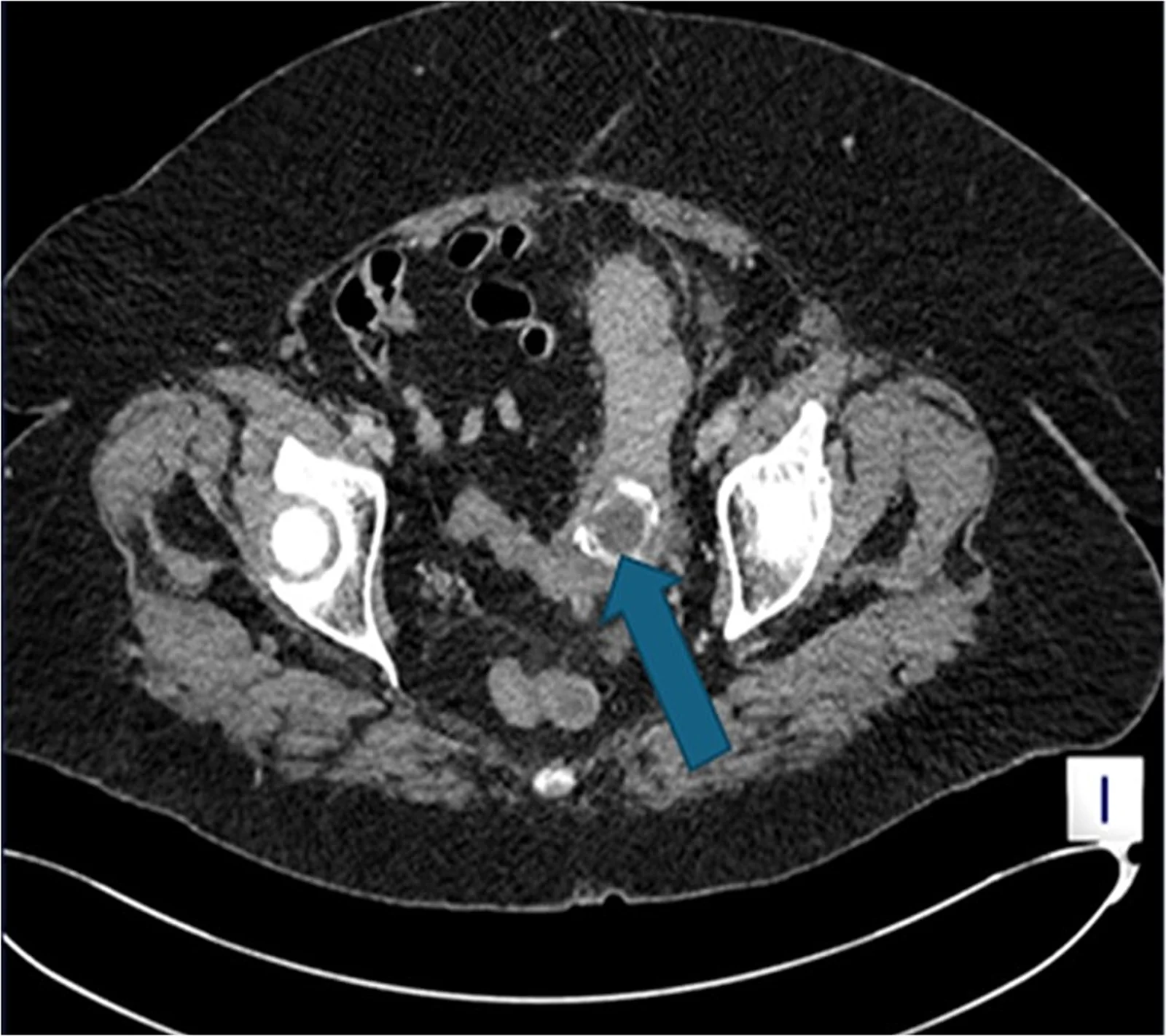
Axial CT image. The low-density gallstone showing some rim calcification is seen in the sigmoid colon (blue arrow).

**Figure 4:**
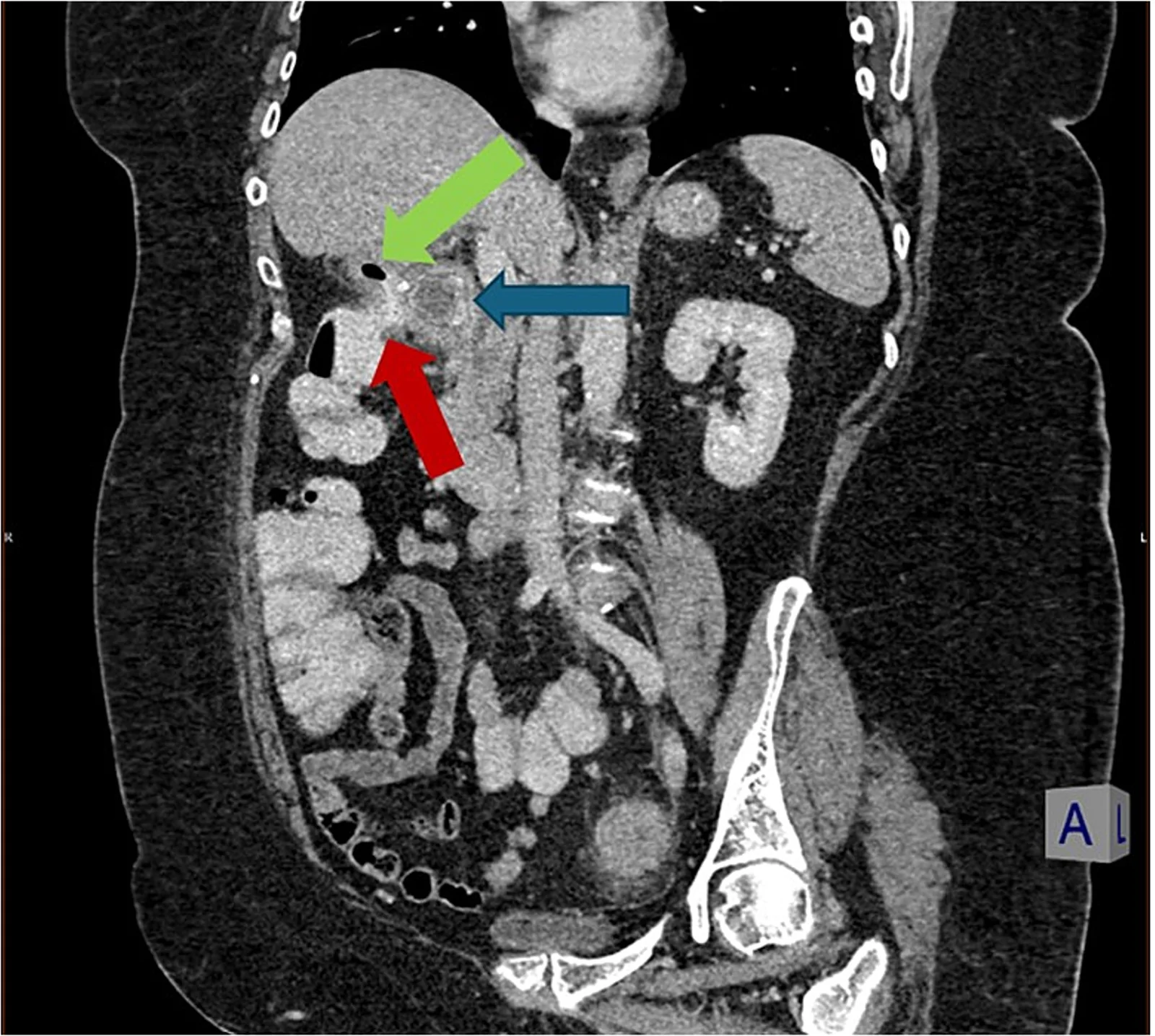
Sagittal reconstruction of CT abdomen with IV and oral contrast. A gallstone is seen in the proximal duodenum (blue arrow). Hyperdense oral contrast outlines the fistulas from the duodenum to the collapsed gallbladder containing air (green arrow) and to the hepatic flexure (red arrow).

Predicted perioperative mortality was 3.7% using NELA and 17.5% using POSSUM. Minimally invasive interventions were therefore attempted. Endoscopic retrograde cholangiopancreatography (ERCP) and push oesophagogastroduodenoscopy failed to dislodge the duodenal stone into the jejunum, while flexible sigmoidoscopic extraction of the sigmoid stone was unsuccessful because of a distal diverticular stricture. Although unconventional, successful endoscopic management has been reported in selected high-risk patients [2].

At laparotomy, proximal jejunotomy enabled extraction of the impacted distal duodenal gallstone (Fig. 5). A Hartmann’s procedure was also performed for the large sigmoid gallstone, which had fistulated through the antimesenteric border proximal to the stricture (Fig. 6).

**Figure 5:**
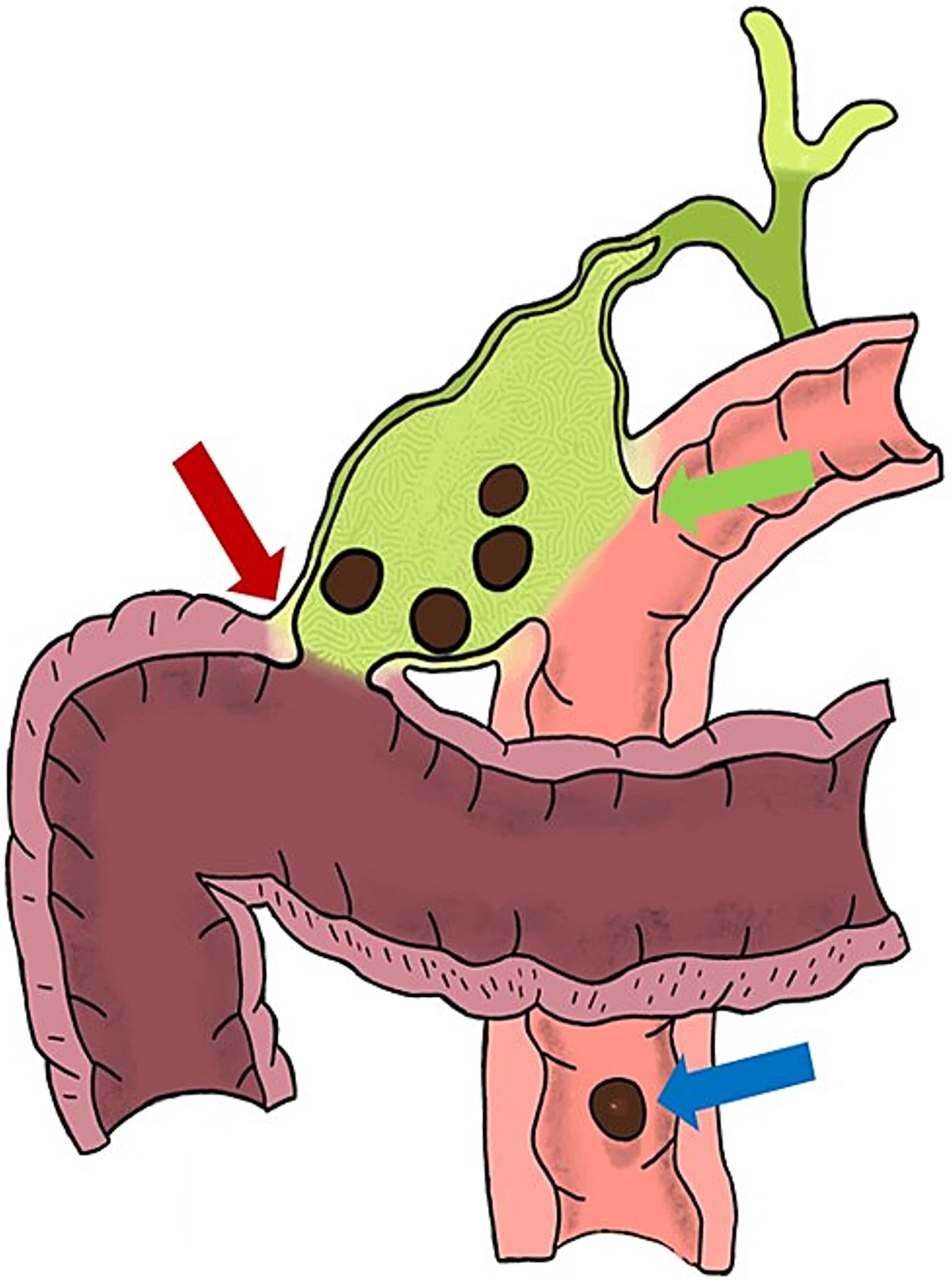
A graphic illustration showing a coronal cross-section of the cholecystocolonic fistula (red arrow), cholecystoduodenal fistula (green arrow), and the gallstone in the proximal duodenum.

**Figure 6:**
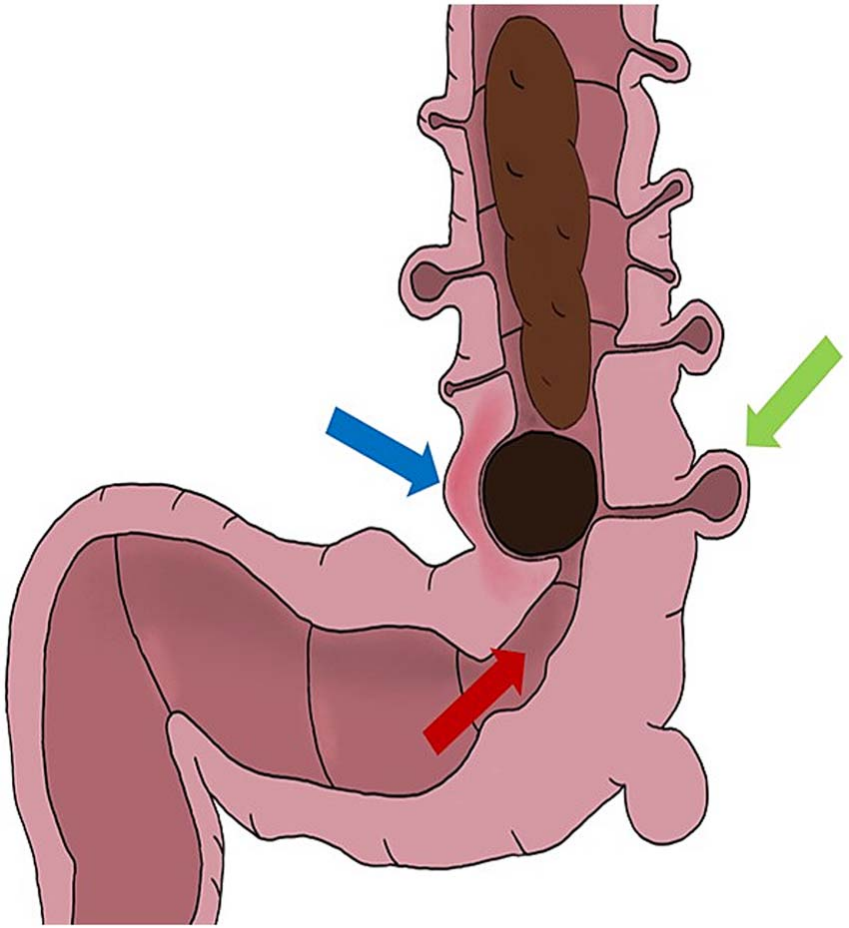
A graphic illustration showing a coronal cross section of the sigmoid colon with diverticula (green arrow), sigmoid stone near fistulating through the antimesenteric border (blue arrow), and the sigmoid stricture (red arrow).

On postoperative day 7, colostomy necrosis and faeculent vomiting necessitated relaparotomy. The colostomy was refashioned, and both cholecystoduodenal and cholecystocolonic fistulae were primarily closed in two layers using 3/0 PDS. Subhepatic and left iliac fossa drains were placed. Cholecystectomy was avoided because the gallbladder was contracted and stone-free. She had a prolonged hospital stay but was safely discharged. Histopathology confirmed benign diverticulosis. At 8-week and 6-month follow-ups, recovery was satisfactory. Hartmann’s reversal was performed one year later, with postoperative CT confirming a patent anastomosis (Fig. 8). The clinical course of her treatment is summarized in Fig. 7.

**Figure 7:**
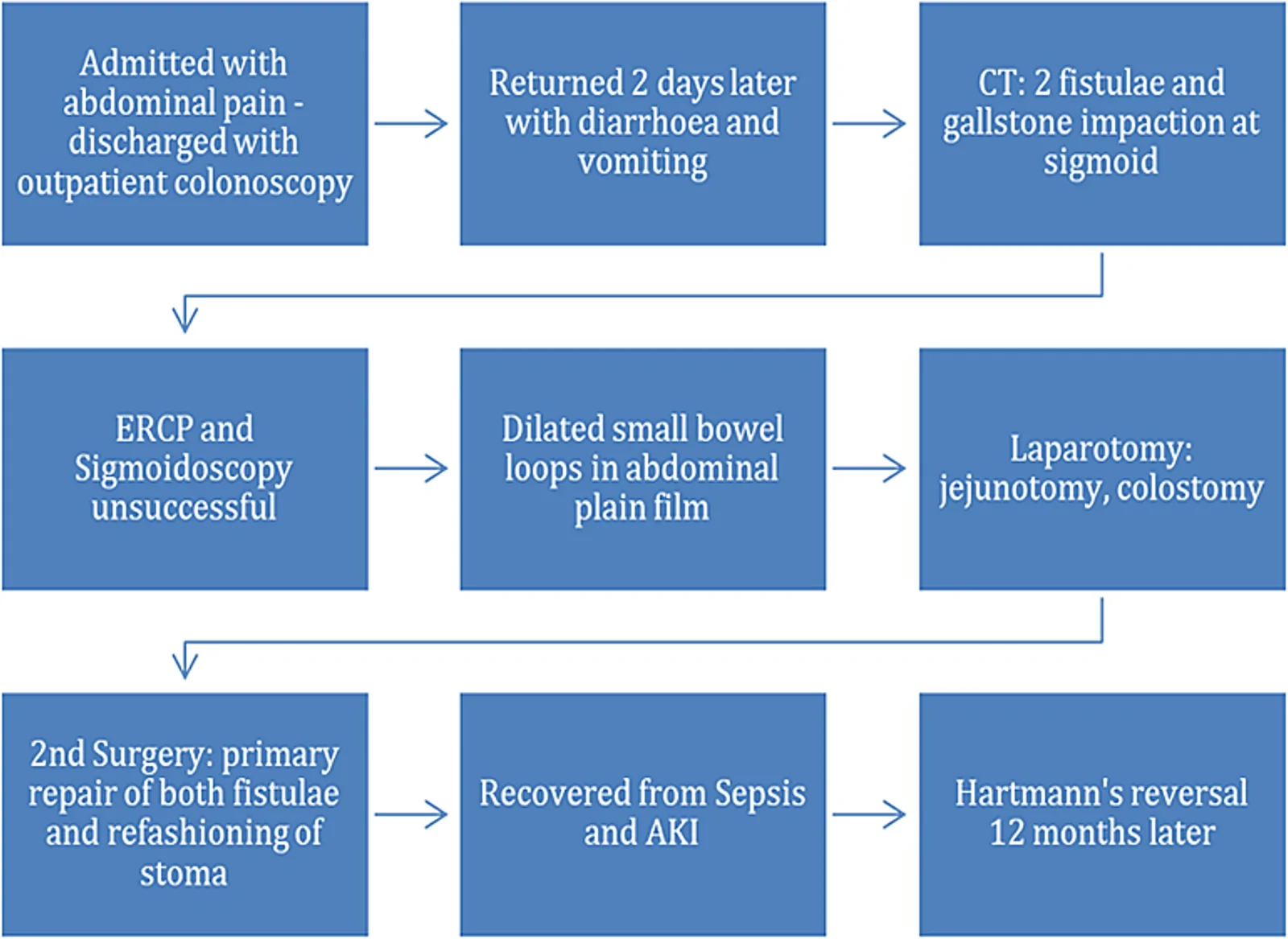
Flowchart showing sequence of events.

**Figure 8:**
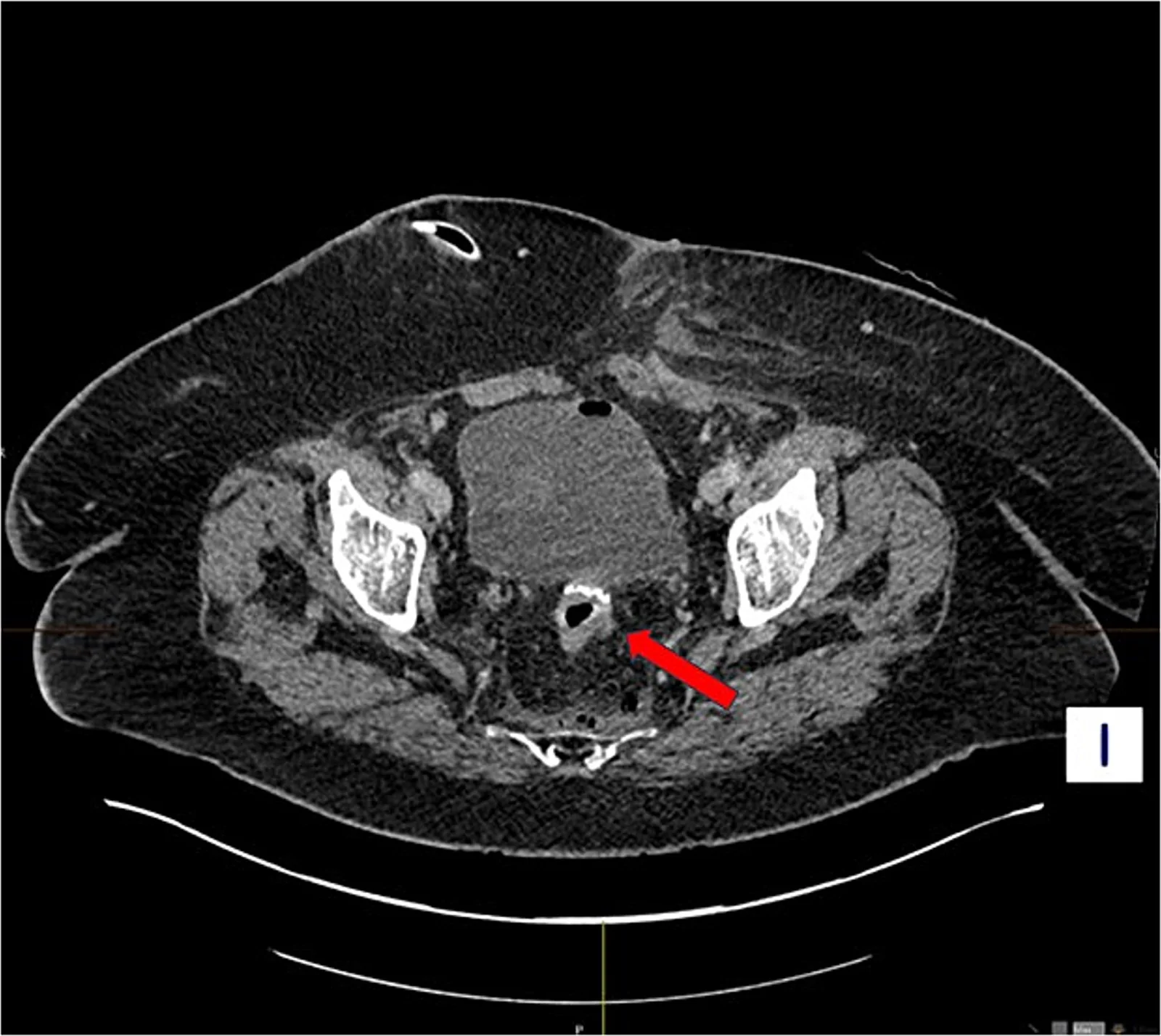
Axial slice demonstrating reversal of Hartmann’s procedure and a patent anastomosis.

## Discussion

In our patient, an impacted gallstone was identified in the sigmoid colon, likely due to a diverticular stricture in this region contributing to the obstruction, further confounding the complexity with CDF. Previous studies report that 59% of patients with sigmoid gallstone ileus also have colonic diverticula, suggesting a potential association between diverticular disease and gallstone impaction [3].

In a study conducted by Glen *et al*., the following was reported: of the 11 808 patients, 0.9% had biliary-enteric fistulae of CDF, accounting for 77%, and CCF was 15% [4]. In a review of 231 cases performed by Costi *et al*., it was reported that 25% presented acutely as biliary ileus [5]. Both CDF and CCF were found in 27 of the 231 cases, making this the most common concomitant abnormality [5]. The optimal radiological modalities for diagnosing CDF and CCF are CT scans, which are readily available during surgical emergencies. Magnetic resonance cholangiopancreatography may help define the anatomy of CDF, but MRIs are not as good as CT for colonic imaging [6].

Treatment options for biliary-enteric fistulas include symptomatic relief, interventional endoscopy, and laparoscopic procedures. The recommended definitive management for biliary enteric fistulas involves cholecystectomy and excision of the fistula [7]. This may be performed laparoscopically or as an open procedure [8].

In this case, a minimally invasive endoscopic approach was initially considered because of the relatively high perioperative mortality, but the trial of both was unsuccessful (endoscopic approach using mechanical or electrohydraulic lithotripsy should still remain a management option [2]. ERCP with biliary sphincterotomy allows for biliary drainage and may aid spontaneous closure of small CDF [6]. In our case, the sigmoid stone was located within endoscopic reach; however, stricturing from the diverticular disease prevented extraction [3]. Studies have shown that impaction at the colonic site often requires surgical intervention and is less likely to close spontaneously [9].

Our patient had a high BMI and previous abdominal surgery, features of impending bowel obstruction and intra-abdominal sepsis; hence, laparotomy was imperative. The chosen staged approach included gallstone removal and treatment of sepsis; Hartmann’s operation resolved the stricture and gallstone fistulation, mitigating the risk of anastomotic leak. This is a safer approach with reduced mortality rates [3, 9]. Staged laparoscopic approach has been advocated by experts when it is safe and feasible to perform for both stages [10].

The development of stoma necrosis expedited the need for a second laparotomy, which allowed for definitive treatment of the fistula and stoma refashioning. This supports a two-stage approach in managing multiple enteric fistulae in the acute setting. Some authors suggested that CDF may close spontaneously once the obstruction has been treated, but closure of the CCF is very uncommon [9].

## Conclusion

Surgical intervention remains the gold standard, performed as a single- or two-stage procedure based on clinical urgency and the patient’s physiological state. Less invasive approaches, such as ERCP and endoscopic stone retrieval, have been suggested for managing CCF and CDF when the patient’s physiological state does not support an invasive approach.

## Data Availability

Not Applicable.

## References

[1] Yu Y, Song Y, Xu J et al. Bouveret's syndrome: a rare presentation of gastric outlet obstruction. Exp Ther Med 2019;17:1813–6. 10.3892/etm.2019.7150 PMC6364238 30783453

[2] Nuño-Guzmán CM, Marín-Contreras ME, Figueroa-Sánchez M et al. Gallstone ileus, clinical presentation, diagnostic and treatment approach. World J Gastrointest Surg 2016;8:65–76. 10.4240/wjgs.v8.i1.65 PMC4724589 26843914

[3] Farkas N, Kaur V, Shanmuganandan A et al. A systematic review of gallstone sigmoid ileus management. Ann Med Surg 2018;27:32–9. 10.1016/j.amsu.2018.01.004 PMC5832643 29511540

[4] Glenn F, Reed C, Grafe WR. Biliary enteric fistula. Surg Gynecol Obstet 1981;153:527–31. https://pubmed.ncbi.nlm.nih.gov/7280941/7280941

[5] Costi R, Randone B, Violi V et al. Cholecystocolonic fistula: facts and myths. A review of the 231 published cases. J Hepatobiliary Pancreatic Surgery 2009;16:8–18. 10.1007/s00534-008-0014-1 19089311

[6] Malik A, Bani Fawwaz BA, Michael M et al. Endoscopic retrograde cholangiopancreatography (ERCP): used to diagnose and treat cholecystoduodenal fistula, a rare clinical entity. Cureus 2021;13:e18962. 10.7759/cureus.18962 PMC8606037 34815904

[7] Ojemolon PE, Kwei-Nsoro R, Haque M et al. Different approaches to the management of cholecystoenteric fistula. ACG Case Rep J 2023;10:e00960. 10.14309/crj.0000000000000960 PMC9857250 36699183

[8] Yokoigawa N, Kawaguchi Y. Preoperative diagnosis and treatment of cholecystoduodenal fistula. Case Rep Gastroenterol 2023;17:249–54. 10.1159/000531486 PMC10624935 37928966

[9] Inukai K . Gallstone ileus: a review. BMJ Open Gastroenterol 2019;6:e000344. 10.1136/bmjgast-2019-000344 PMC6904169 31875141

[10] Huang SF, Han YH, Chen J et al. Surgical management of cholecystoenteric fistula in patients with and without gallstone ileus: an experience of 29 cases. Front Surg 2022;9:950292. 10.3389/fsurg.2022.950292 PMC9304664 35874133

